# Development of a Highly Sensitive and Specific ic-ELISA and Lateral Flow Immunoassay for Diacetoxyscirpenol

**DOI:** 10.3390/foods11111548

**Published:** 2022-05-25

**Authors:** Shibei Shao, Wenhua Shang, Yuchen Bai, Leina Dou, Suxia Zhang, Jianzhong Shen, Zhanhui Wang, Kai Wen

**Affiliations:** Beijing Key Laboratory of Detection Technology for Animal-Derived Food Safety, Beijing Laboratory for Food Quality and Safety, College of Veterinary Medicine, China Agricultural University, Beijing 100193, China; shaoshibei@cau.edu.cn (S.S.); s20213050793@cau.edu.cn (W.S.); BS20193050465@cau.edu.cn (Y.B.); b20193050410@cau.edu.cn (L.D.); suxia@cau.edu.cn (S.Z.); sjz@cau.edu.cn (J.S.); wangzhanhui@cau.edu.cn (Z.W.)

**Keywords:** diacetoxyscirpenol, trichothecenes, monoclonal antibody, ELISA, LFIA, antibody molecular recognition

## Abstract

To monitor the contamination of a type A trichothecene, diacetoxyscirpenol (DAS), one monoclonal antibody (mAb) 8A9 with high affinity and specificity was prepared in the present study. The mAb 8A9 showed a 50% inhibition concentration (IC_50_) of 0.31 μg/L, which is of the highest affinity reported to date. An indirect competitive enzyme-linked immunosorbent assay (ic-ELISA) and lateral flow immunoassay (LFIA) based on mAb 8A9 were developed and exhibited limits of detection as low as 0.65 μg/kg and 100 μg/kg in rice samples, respectively. The molecular recognition mechanism of mAb 8A9 to DAS was explored by molecular docking. The results showed that the hydrophobic amino acids of mAb 8A9 interacted with DAS by forming hydrogen bonds and a pi-sigma bond, which lead to a highly specific recognition of DAS. In summary, we produced one mAb, developed ELISA and LFIA for DAS detection in rice with significantly sensitivity, specificity, accuracy, and precision.

## 1. Introduction

**Figure 1:**
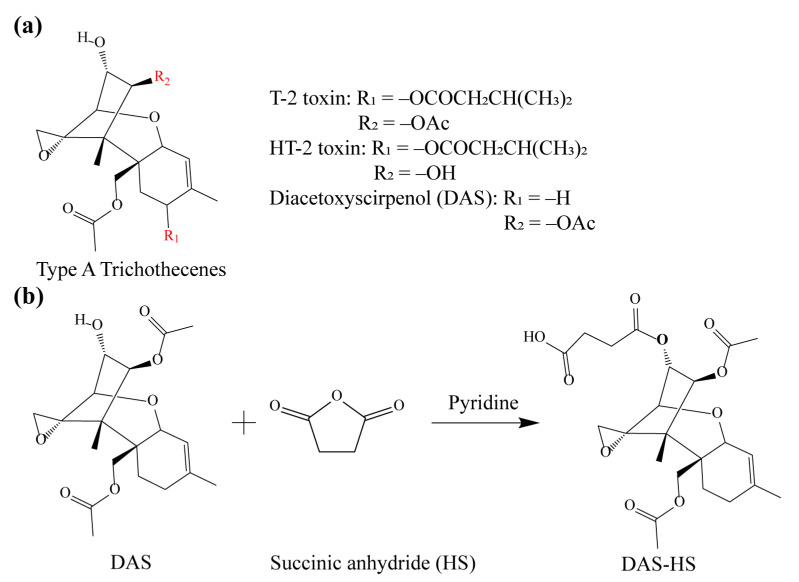
Structures of DAS and hapten. (**a**) Structures of type A trichothecenes, (**b**) synthetic route of DAS hapten.

Diacetoxyscirpenol (DAS) is a mycotoxin of type A trichothecenes as shown in Figure 1a, which are highly toxic secondary metabolites produced by *Fusarium* fungi [1,2,3]. As with T-2 toxin and HT-2 toxin in the type A trichothecene group, exposure of DAS causes potential risks for animal and human health. The intake of DAS may induce immunotoxicity [4,5], hematotoxicity [6], growth retardation [7], digestive tract diseases [8,9], pulmonary disorders [10], and cardiovascular effects [11] by inhibiting the synthesis of protein and DNA [12,13]. DAS also has the potential of triggering hypoxia in cells [14] and resulting in a significant reduction in somatic and germ cells [4,12]. DAS was found in many feeds, cereals, and foods [15], mainly in African, American, and European countries [16,17,18,19]. Therefore, the establishment of highly sensitive and specific assays for DAS determination is essential to ensure food safety and human health.

Analytical methods for determination of DAS in foods have been developed, such as LC-MS/MS [20,21], GC-MS [22,23,24], thin-layer chromatography-mass spectrometry [25], and UPLC-MS [26]. Instrumental methods can facilitate the confirmatory analysis of DAS in food. However, there are challenges for less developed regions because of the high cost of instruments and the need for skilled operation, as well as the lack of applicability in on-site monitoring. Immunoassays are becoming popular as a simple and cost-effective way to detect contaminants in food samples [27,28].

Immunoassays based on antigen–antibody recognition have been reported for screening DAS. Researchers have prepared rabbit polyclonal antibodies for the development of radioimmunoassay or indirect competitive enzyme-linked immunosorbent assay (ic-ELISA) to detect DAS, which were not sufficiently sensitive with a 50% inhibition concentration (IC_50_) of above 10 μg/L, and had cross-reactivity (CR) of 5–500% to T-2 toxin and other trichothecenes [29,30,31,32,33]. Monoclonal antibody (mAb) produced from mice have also been reported to detect DAS, however, the obtained mAbs showed low affinity to DAS with IC_50_ over 10 μg/L [34], which are insufficient to develop highly sensitive immunoassays for DAS. Jin et al. [35] immunized mice to obtain an mAb with IC_50_ of 5.97 μg/L and established ic-ELISA and immunochromatographic methods for DAS detection in rice samples with a detection limit of 0.78 μg/L and a cut-off value of 500 ng/g, respectively, which was not sensitive enough for detection because of the susceptibility to sample matrix interference. Tang et al. [36] prepared one mAb with IC_50_ of 3.08 μg/L and established a competitive pressure-dependent immunosensor for the highly sensitive detection of DAS in wheat, but the method needed an electrochemical workstation, which caused a cost burden for rapid detection. Therefore, it is necessary to prepare mAb with specificity and high affinity to construct immunoassays for the detection of DAS.

In this work, we prepared one new mAb against DAS with the highest affinity. Highly sensitive ic-ELISA and rapid lateral flow immunoassay (LFIA) were established based on the mAb to determine DAS. Recovery studies in rice were used to assess the performance of the developed ic-ELISA. Furthermore, the variable regions of mAb were established to clarify the recognition mechanism to DAS. The constructed methods could fill the gap of highly sensitive and rapid immunoassay for DAS detection.

## 2. Materials and Methods

### 2.1. Reagents and Materials

DAS, T-2 toxin, HT-2 toxin, aflatoxin B_1_, citreoviridin, deoxynivalenol, fumonisin B_1_, zearalenone, and ochratoxin A were purchased from J&K Chemical Technology (Beijing, China). N-hydroxysuccinimide (NHS), 1-ethyl-carbodiimide hydrochloride (EDC), pyridine, 4-dimethylaminopyridine (DMAP), succinic anhydride (HS), carbonyldiimidazole (CDI), bovine thyroglobulin (BTG), bovine serum albumin (BSA), complete and incomplete Freund’s adjuvant, hypoxanthine aminopterin thymidine (HAT), and poly (ethylene glycol) (PEG) 1500, were acquired from Sigma-Aldrich (St. Louis, MO, USA). Cell culture medium (DMEM) was bought from Thermo Fisher Scientific (Waltham, MA, USA). Horseradish peroxidase (HRP)-conjugated goat anti-mouse IgG was purchased from Jackson Immuno Research (West Grove, PA, USA). Fetal bovine serum was obtained from Gibco BRL (Carlsbad, CA, USA). TMB (3,3′,5,5′-tetramethyl benzidine) substrate solution and hydrogen peroxide (H_2_O_2_) were purchased from Beyotime (Shanghai, China). Other analytical-grade pure reagents were bought from National Pharmaceutical Group Chemical Reagents Co., Ltd. (Beijing, China). Distilled water was obtained from a Milli-Q purification system (Bedford, MA, USA). Microplates for ELISA were acquired from Costar (Cambridge, MA, USA). Polystyrene cell culture plates were obtained from Corning Life Sciences (New York, NY, USA). Nitrocellulose (NC) membrane was purchased from Millipore Corp. (Bedford, MA, USA). Polyvinylchloride (PVC) backing materials, sample pad, and absorbance pad were purchased from Goldbio Tech Co. (Shanghai, China). Balb/c mice were supplied by Beijing Vital River Laboratory Animal Technology (Beijing, China). The ZX1000 dispensing platform and the CM4000 guillotine cutting module was purchased from BioDot, Inc. (Irvine, CA, USA). The buffer and solution used in this work can be found in the Supplementary Materials.

### 2.2. Synthesis of Hapten and Antigen

The synthetic route of DAS hapten has been shown in Figure 1b. DAS (1.0 mg) was dissolved in pyridine, then succinic anhydride (7.2 mg) and DMAP (3.4 mg) were added. The mixture was stirred on a magnetic stirrer at 50 °C for 5 h. Pure water (50 μL) was added to quench the reaction and the mix was then dried under nitrogen flow. The residue was extracted with pure water and trichloroethane. The organic phases were dried under nitrogen to obtain hapten DAS-HS.

DAS-HS (1.0 mg), NHS (1.0 mg), and EDC (1.7 mg) were dissolved in DMF (500 μL) and stirred at room temperature for 6–8 h. After BTG (10.0 mg) was dissolved in 0.05 M CB (5 mL), the above DMF reaction was added dropwise to the BTG solution under ice bath. The product was transferred to a dialysis bag and dialyzed with PBS for 3 days (the dialysis solution was changed every 8 h). Finally, the product of immunogen DAS-HS-BTG was obtained.

Similarly, the active hydroxyl group of DAS was used to couple with BSA by CDI method to prepare the coating antigen. DAS (1.0 mg) was dissolved in DMF (0.2 mL) and CDI (8.0 mg) was added to the solution with magnetic stirring at 37 °C for 2 h. BSA (10.0 mg) was dissolved in 0.05 M CB (5 mL) and the above activation solution was slowly added dropwise to the protein solution under ice bath with stirring overnight. The reaction product was transferred to a dialysis bag and dialyzed with PBS for 3 days (the dialysis solution was changed every 8 h). The obtained product is coating antigen of DAS-CDI-BSA.

### 2.3. Production of mAb

All animal experiments were conducted in strict accordance with Chinese laws and guidelines approved by the animal ethics committee of China Agricultural University. Eight Balb/c female mice aged 8 weeks were immunized with immunogen (diluted in PBS to 1.0 mg/mL) at a dose of 100 μg per mouse on an identical schedule. Mice were injected subcutaneously with an emulsified mixture of equal volumes complete Freund’s adjuvant and immunogen for primary immunization. One month after the first immunization, mice were immunized with an emulsified mixture of incomplete Freund’s adjuvant and immunogen for twice the enhancement every 3 weeks. The 4th immunization was given via intraperitoneal injection without the adjuvant. Serum of immunized mice was collected 7 days after each immunization and determined using ic-ELISA. Four days after the last immunization, mice spleen cells were separated and fused with PEG 1500 pretreated sp2/0 myeloma cells to prepare hybridomas according to procedures described previously [37]. The fused cells were cultured in HAT medium for 7 days and the supernatant was determined using ic-ELISA. The highly sensitive hybridomas were retained after subcloning four times. Finally, the hybridomas were intraperitoneally injected into mice, and the ascites collected from mice were extracted and purified with saturated ammonium sulfate to obtain purified mAbs.

### 2.4. Development and Optimization of ic-ELISA

Microtiter plates were coated with 100 μL coating antigen DAS-CDI-BSA (0.25 μg/mL in coating buffer), and incubated at 4 °C overnight. The plates were washed three times with washing buffer, and then blocked in 150 μL/well blocking buffer at 37 °C for 2 h. After blocking, 50 μL of analytes dilutions and 50 μL of diluted mAb were added and incubated at 37 °C for 30 min. Then the plates were washed three times and goat anti-mouse IgG-HRP (1/5000 in PBS, 100 μL/well) was added, and incubated at 37 °C for 30 min. Following incubation, the plates were washed three times, and then substrate solution (100 μL/well) was added and incubated at 37 °C for 15 min. Fifty microliters per well of 2 M H_2_SO_4_ was added to stop the reaction. The optical density (OD) value at 450 nm of each well was measured using an ELISA microplate reader.

Eight toxins were used to evaluate the CR of the antibody. Standard curves were prepared in assay buffer and each IC_50_ value was determined in the competitive experiment described above. The CR values were calculated according to the following equation [37]:

CR = IC_50_ (DAS, μg/L)/IC_50_ (analogs, μg/L) × 100% (1)

The concentrations of coating antigen and antibody were optimized using a checkerboard procedure and then pH value (5.0, 6.0, 7.0, 8.0, 9.0, 10.0, 11.0, and 12.0), ionic strength (concentrations of NaCl were 0.01, 0.02, 0.04, 0.08, 0.2, 0.4, 0.8, 1.0, 2.0, and 4.0 mol/L), and organic solvent acetonitrile (ACN, proportion was 1%, 2%, 5%, 10%, 20%, and 40%) of assay buffer were optimized. The parameter A_max_ (the OD value of negative well)/IC_50_ was introduced here as evaluation index, and the larger value means the higher affinity of mAb.

### 2.5. Matrix Effect and Recovery

Rice samples was purchased at a local supermarket. Two grams of homogenized rice sample were weighed and DAS of different concentrations was added. Each sample was then extracted with 8 mL of 25% ACN-PBS (4-fold dilution) and was oscillated on a shaker for 0.5 h and then centrifuged at 5000× *g* for 10 min. The supernatant was diluted 10 times in PBS for ic-ELISA and LFIA analysis. The parallelism of the sigmoidal curves was compared to that prepared in the pure assay buffer in order to evaluate the extent of the interferences caused by the matrix. The limit of detection (LOD) and recovery served as criteria to evaluate the ic-ELISA. LOD of ic-ELISA was determined as the mean concentration of 20 blank rice samples plus three times the standard deviation. A recovery test was used to assess the accuracy of the ic-ELISA. Briefly, blank samples were spiked to three different final concentrations of DAS (50/100/200 μg/kg), and then the spiked samples were pretreated and subjected to ic-ELISA for recovery analysis. The recovery tests were performed in three independent replicates. Cut-off value served as criteria to evaluate LFIA, blank samples were spiked with DAS to serial final concentrations (0/6.25/12.5/25/50/100/200/400 μg/kg), respectively, and then the spiked samples were pretreated as above and subjected to LFIA.

### 2.6. Preparation of mAb-Labeled Colloidal Gold Nanoparticles (AuNPs)

AuNPs with radius of 30 nm were synthesized according to previous reports [38]. The pH value of AuNPs was adjusted with 0.1 M K_2_CO_3_, and then anti-DAS mAb was added and mixed rapidly. After reacting for 10 min at room temperature, 20 μL of 20% BSA solution (*w*/*v*) was added and incubated for 15 min. The mixture was centrifugated at 8000× *g* for 10 min, the supernatant was discarded and the precipitation was resuspended in 0.02 M PBS (containing 1% BSA, pH 7.4).

### 2.7. Preparation of Lateral Flow Immunoassay Strip

Nitrocellulose (NC) membrane was sprayed with coating antigen DAS-CDI-BSA and goat anti-mouse IgG antibody, respectively, to form the test (T) line and control (C) line with a rate of 0.8 μL/cm using a membrane dispenser, and then dried at 37 °C overnight. A sample pad, a dried NC membrane, and an absorbent pad were assembled layer by layer with 1 mm overlap on a PVC backing card, and the assembled card was cut into 4 mm wide strips, and stored in a moisture-proof box at room temperature.

Ten microliters of mAb-labeled AuNP was mixed with 190 μL of PBS containing DAS in microwells and incubated for 5 min at 37 °C. The mixed solution was added to the sample pad to flow through the strip.

### 2.8. Extraction of Antibody Variable Region Sequences, Homology Modeling and Molecular Docking

Hybridoma cells (1 × 10^7^) of 8A9 were collected and lysed to extract total RNA according to the technical manual of the RNeasy Plus Micro Kit (Qiagen Japan Co., Ltd., Tokyo, Japan). Then, the isolated RNA was reverse-transcribed into cDNA using either isotype-specific antisense primers of SMART Scribe Reverse Transcriptase (Clontech, Tokyo, Japan). Variable fragments of heavy chains and light chains of antibody were then amplified by PCR. The primers for amplification are listed in Table S1, and PCR procedure was 30 cycles of 95 °C, 60 s; 65 °C, 30 s; and 72 °C, 120 s. Amplified antibody fragments were cloned into standard cloning vectors and followed by a colony PCR to screen for clones. After clones were incubated in LB liquid medium overnight at 37 °C and 250 rpm, the obtained bacteria liquid was sent to Genewiz (Suzhou, China) for Sanger sequencing. The frame region and complementarity determining regions (CDR) of single-chain antibodies (scFv) were clarified using the IMGT numbering method. Three-dimensional structure predictions for scFv were generated by Model Antibody in Discovery Studio 2019 program (BIOVIA Corp., San Diego, CA, USA) based on the sequence of scFv. According to the sequence similarity and identity, the best matching overall template was used to model antibody framework. The binding between scFv and DAS was performed using CDOCKER in Discovery Studio 2019. 

## 3. Results and Discussion

### 3.1. Preparation and Identification of Hapten and Antigen

The DAS-HS haptens were purified and identified by positive-ion mass spectrometry; the correct mass of the main product with molecular weight of 467 as shown in Figure S1a demonstrated the successful synthesis of DAS-HS. DAS-HS (or DAS) was conjugated with BTG (BSA) by carbodiimide method (or CDI method) to form immunogen (or coating antigen). The BTG conjugates were characterized by UV-vis spectrum (Figure S1b), and the BSA conjugates were characterized using matrix-assisted laser desorption/ionization time of flight mass spectrometry (Figure S1c,d). The peaks of DAS-HS-BTG appear significantly red-shifted relative to the BTG standard, and blue-shifted relative to DAS-HS, indicating successful synthesis of the DAS-HS-BTG immunogen. The DAS-CDI-BSA and the standard of BSA showed significant mass discrimination, demonstrating successful synthesis of the complete antigen.

### 3.2. Production of mAb to DAS

DAS-HS-BTG was used as immunogen to immunize eight six-week-old female BALB/c mice (100 μg/mice), and DAS-CDI-BSA was used as coating antigen for screening. A total of 8 clones were eventually screened out. The clones with the best titer and inhibition (3F10, 4F8, 8A9) were identified by ic-ELISA (Figure S2a), and mAb 8A9 exhibited the lowest IC_50_ and was finally selected for mAb production. Besides, the size distribution and thermo-stability of mAb 8A9 were evaluated in this work. Results showed that the particle size distribution of mAb 8A9 in PBS buffer was homogeneous with an average particle size of 11.9 nm. The aggregation temperature (T_agg_) and denaturation temperature (T_m_) of mAb 8A9 were 72.4 °C and 75.5 °C, respectively, measured by UNcle (Unchained Labs, Pleasanton, CA, USA). The results demonstrated that the mAb 8A9 exhibited excellent thermo-stability and was beneficial to establish robust assay in complex food matrixes (Figure S2b,c).

### 3.3. Optimization and Development of ic-ELISA

**Figure 2:**
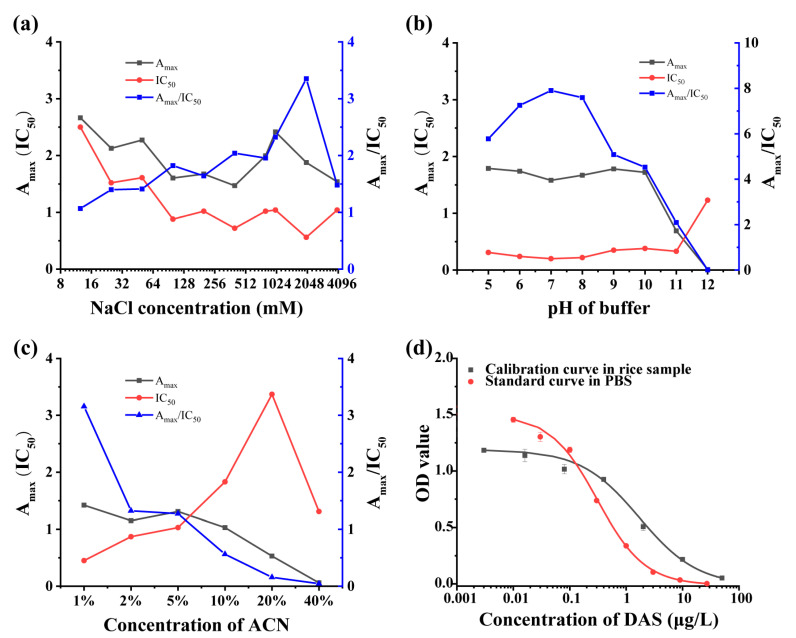
Optimization of physicochemical parameters. (**a**) NaCl concentration, (**b**) pH value, and (**c**) concentration of ACN on the ic-ELISA and the calibration curves assessed by the ic-ELISA (*n* = 3). (**d**) Standard curve of ic-ELISA based on mAb 8A9 for DAS in PBS buffer and calibration curve in the diluted rice sample (*n* = 3).

Physicochemical factors that influence the performance of ic-ELISA were evaluated under the optimal concentrations of coating antigens (0.3 μg/mL), mAb (1 μg/mL), and goat anti-mouse IgG (1/5000), including NaCl concentrations, pH value, and organic solvent. It can be seen in Figure 2a that the A_max_/IC_50_ ratio expressed a numerical increase with increasing ionic strength below 4 M NaCl, which showed that the mAb provided a high stability to ionic strength. The ratio of A_max_/IC_50_ was higher at pH 7.0–8.0 (Figure 2b), the mAb was relatively stable with pH changes in assay environment. Additionally, the results of organic solvent test indicated that the A_max_/IC_50_ ratio decreased significantly when the concentration of acetonitrile is over 5% (Figure 2c). These results indicated that the mAb could not tolerant acetonitrile, thus PBS without organic solvent was chosen as assay buffer for later experiments. A standard curve of ic-ELISA was determined for DAS (Figure 2d). The IC_50_ and linear range (IC_20_–IC_80_) of the developed ic-ELISA were 0.31 μg/L and 0.08–1.18 μg/L in buffer, respectively. The method shows 10-fold lower IC_50_ to that of previous studies (Table 1).

The CR of the mAb 8A9 to analogs is shown in Table 2; the antibody recognized DAS with highly affinity but did not exhibit measurable CRs with other mycotoxins.

### 3.4. Matrix Effect and Recovery of ic-ELISA

Blank rice sample extracts were used here to evaluate matrix effect. As shown in Figure 2d, the IC_50_ values of ic-ELISA for DAS were 0.31 μg/L in PBS and 1.71 μg/L in 40-fold diluted rice extracts. There was at least 5-fold increase in IC_50_ value even after 40-fold dilution, indicating that the matrix effect cannot be ignored by dilution strategy, and matrix calibration curve for the detection of DAS in sample was employed. The LOD of the ic-ELISA for DAS was determined as 0.65 μg/kg in rice by calculating the mean concentration of 20 blank samples plus three times the standard deviation.

To evaluate the accuracy of the established ic-ELISA, recovery and coefficient of variation (CV) of spiked rice samples were measured. DAS at concentrations of 50, 100, and 200 μg/kg were spiked into blank samples. As shown in Table 3, the recoveries ranged from 95 to 124% with CV less than 14%, which indicates the reliability of the ic-ELISA for DAS detection in rice sample.

### 3.5. Development of LFIA

**Figure 3:**
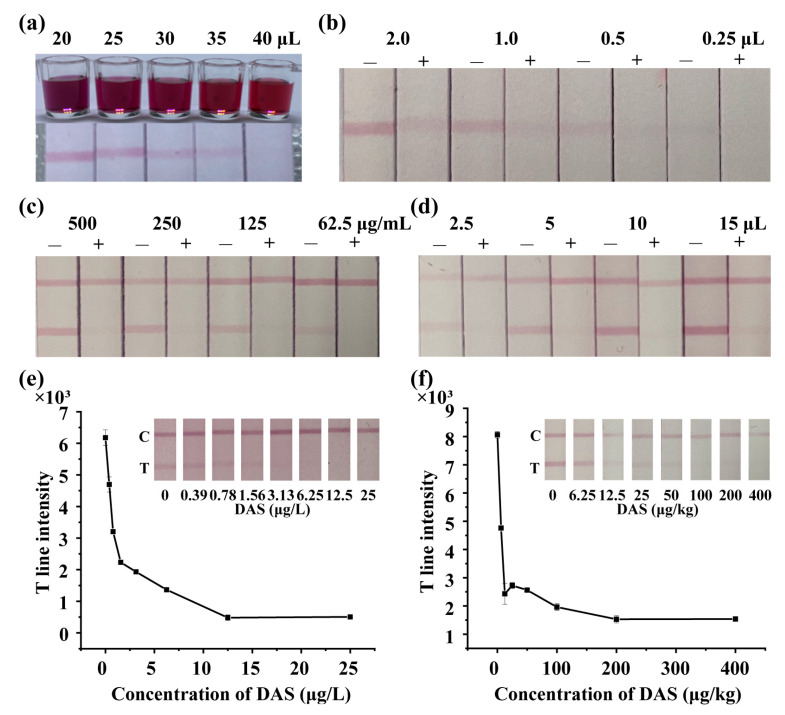
Optimization and detection of the LFIA strips. Optimization of (**a**) the volume of K_2_CO_3_, (**b**) amount of mAb, (**c**) concentration of coating antigen and (**d**) amount of mAb-labelled AuNPs with a negative sample (0 μg/L DAS) and a positive sample (25 μg/L DAS). “−” means negative, “+” means positive. Various concentrations of DAS in (**e**) PBS buffer and (**f**) rice samples have been detected.

According to the results of ic-ELISA, DAS-CDI-BSA could be used as the coating antigen to develop LFIA. AuNPs were synthesized and characterized by UV-vis spectrum and transmission electron microscopy (Figure S3). Parameters such as the labelling pH, the amount of labelling mAb, the coating antigen concentration, and the amount of mAb-labelled AuNPs have been optimized with negative sample (0 μg/L DAS) and positive sample (25 μg/L DAS). As shown in Figure 3a, the pH (volume of K_2_CO_3_ used) of AuNPs can influence the color intensity of the T line of LFIA significantly. When less than 25 μL K_2_CO_3_ is used, it might cause instability of the labelled complex, which means strong aggregation of negative electrical AuNPs due to a pH lower than pI of the mAb, thus 25 μL of K_2_CO_3_ was chosen for the following experiment. As shown in Figure 3b,c, the color intensity of the T line decreased as the amounts of mAb and coating antigen decreased. However, too much mAb or coating antigen might cause unspecific signal, thus the optimal mAb amount and coating antigen concentration were 1.0 μL and 250 μg/mL, respectively. The effect of different amounts of mAb-labelled AuNPs is shown in Figure 3d. It was obvious that the color of the T line of the negative sample was deep while that of the positive sample was clean when the amount of mAb-labelled AuNPs was 10 μL, thus 10 μL labelled complex was used in later experiments.

Based on the optimal conditions, DAS with various concentrations were spiked into PBS buffer and rice samples to determine the cut-off value of the LFIA. As shown in Figure 3e,f, the cutoff value of the LFIA strip was 6.25 μg/L in PBS and 100 μg/kg in rice samples.

### 3.6. Recognition Mechanism of mAb to DAS

**Figure 4:**
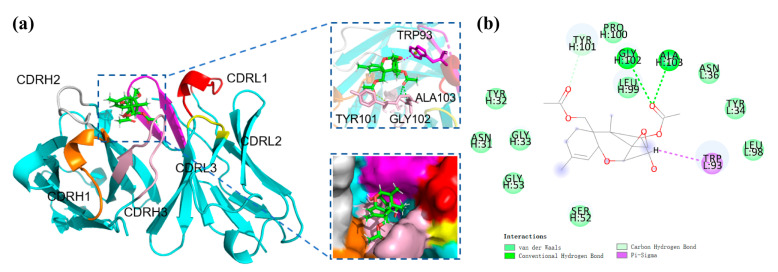
Molecular docking of the scFv–DAS interaction. (**a**) Molecular docking of scFv 8A9 and DAS, (**b**) 2D views of DAS interacting with the scFv. The red, yellow, and purple components are CDRL1, L2, and L3, respectively; and the orange, silver, and pink components are CDRH1, H2, and H3, respectively. Hydrophobic interactions are shown as purple dashes and hydrogen bonds are displayed as green dashed lines.

The mAb obtained in this work showed higher affinity and specificity than previous ones. To explain the recognition mechanism of mAb 8A9 to DAS, structural studies on DAS in complex with the scFv 8A9 were conducted. The modeled scFv 8A9 were docked with DAS as shown in Figure 4a, and the 2D views of DAS interacting with the scFv is shown in Figure 4b. It is generally believed that CDR3 are the centers of traditional antigen binding sites [39]. As shown in Figure 4a, the binding pocket of antibody is mainly composed of the CDRL3, CDRL1, and CDRH3 regions of the scFv. Amino acid residues with hydrophobic side chains (PRO, TYR, GLY, LEU, ALA) are distributed around the DAS molecule (Figure 4b) forming a compact hydrophobic pocket covering the DAS surface. Meanwhile, the parent nucleus structure of DAS is deeply inserted into the binding cavity. As shown in Figure 4b, four key amino acids of scFv 8A9, TYR101, GLY102, ALA103, and TRP93, form two hydrogen bonds and a pi-sigma bond with DAS. The relative stronger pi-sigma bond formed by TRP93 in CDRL3 with DAS near the edge of the binding pocket might contribute the high affinity of antibody to DAS. We conjectured that during the binding process, the R1 side chain moiety of DAS firstly entered the binding pocket under hydrophobic forces and hydrogen bonding forces, and the parent nucleus moiety of DAS subsequently reached the binding pocket and was caught by strong pi-sigma bond formed with the binding pocket. Antibodies in previous studies showed significant CR to T-2 toxin and HT-2 toxin [29,30,31,32], but the mAb 8A9 in the present study hardly recognized these two type A trichothecenes, which may be related to the fact that the binding pocket of DAS-induced mAb in the present study is small and fits closely to the structure of the DAS molecule. Since the R1 moiety is the primary structure during binding by the compact hydrophobic pocket of antibody 8A9, the R_1_ group (-OCOCH_2_CH(CH_3_)_2_) in both T-2 toxin and HT-2 toxin is relative larger than that of DAS (-H) (Figure 1a), resulting in a steric hindrance and the inability to enter the binding pocket of antibody 8A9 and thus cannot be recognized by antibody 8A9. In this context, despite that the trichothecenes have identical parent nucleus moiety, the structural differences in key R1 group recognized by antibody led to the differences in antibody recognition, which means that the difference in the structure of side chain still has an important effect on the formation of the binding cavity.

## 4. Conclusions

In this study, one new mAb against DAS with high affinity and specificity was obtained, of which the affinity was more than ten times higher than previous studies, and it was highly specific, with a very low rate of cross-reactivity to other microbial toxins. The ic-ELISA and LFIA based on the mAb for screening DAS in rice with significantly improved sensitivity and specificity were established, the detection limit and cut-off value could be more than 5 times lower than previous studies. Furthermore, the recognition mechanism of antibody to DAS was analyzed, the results showed that its high affinity may be related to the fact that the shape of the binding pocket closely fits the shape of DAS, and the relatively strong pi-sigma bond formed between the hydrophobic binding pocket and DAS, which leads to the inability of homologous trichothecenes with the same parent nucleus moiety but different side chain structures to be recognized by the antibody, thus resulting in its high specificity. The preparation of DAS antibody and the establishment of rapid detection methods provide excellent reagents for the detection of DAS and fill the gap of sensitive immunoassay of DAS. It provides the possibility to detect the co-contamination of multiple toxins in food, and also lays the foundation for the formulation of its limit standards in the future, thus ensuring food safety and human health.

## Figures and Tables

**Table 1:** Reported antibodies for determination of DAS.

Antibody Type	IC_50_ (μg/L)	Immunoassays	Reference
pAb	15	RIA	[29]
pAb	Not mentioned	ic-ELISA	[30]
pAb	10	ic-ELISA	[31]
pAb	Not mentioned	ic-ELISA	[32]
pAb	Not mentioned	ic-ELISA	[33]
mAb	Not mentioned	RIA	[34]
mAb	5.97	ic-ELISA	[35]
mAb	3.08	competitive-type pressure-dependent immunosensor	[36]
mAb	0.31	ic-ELISA	This study

**Table 2:** The cross-reactivity (CR) values of the ELISA for detecting DAS and analogs.

Analyte	IC_50_ (μg/L)	Cross-Reactivity (%)
DAS	0.31	100
T-2 toxin	>5000	<0.1
HT-2 toxin	>5000	<0.1
Aflatoxin B_1_	>5000	<0.1
Fumonisin B_1_	>5000	<0.1
Deoxynivalenol	>5000	<0.1
Citreoviridin	>5000	<0.1
Ochratoxin A	>5000	<0.1
Zearalenone	>5000	<0.1

**Table 3:** The recoveries of the ic-ELISA for the determination of DAS in rice.

Spiked Level (μg/kg)	Mean Recovery (%)	Inter-Assay CV (%)	Intra-Assay CV (%)
50	114	10	4
	106	7	
	109	13	
100	95	12	14
	120	12	
	124	4	
200	96	12	5
	106	13	
	104	7	

## Data Availability

The data presented in this study are available in this article and supplementary materials.

## Supplementary Materials

The following supporting information can be downloaded at: https://www.mdpi.com/article/10.3390/foods11111548/s1, Figure S1: Characterizations of DAS hapten and antigen; Figure S2: Characterizations of DAS mAb; Figure S3: Characterizations of AuNPs; Table S1: Primers used for PCR amplification.
