# Supplementary material for: Development of a Highly Sensitive and Specific ic-ELISA and Lateral Flow Immunoassay for Diacetoxyscirpenol
Source: Foods. 2022 May 25;11(11):1548. doi: 10.3390/foods11111548 (PMC9180083; doi:10.3390/foods11111548)
Supplement: Supplementary file 1 [file foods-11-01548-s001.zip › foods-1709637-supplementary.pdf]

Supplementary Materials

Development of a highly sensitive and specific ic-ELISA and a lateral flow immunoassay for diacetoxyscirpenol

Shibei Shao, Wenhua Shang, Yuchen Bai, Suxia Zhang, Jianzhong Shen, Zhanhui Wang and Kai Wen*

Beijing Laboratory for Food Quality and Safety, Beijing Key Laboratory of Detection Technology for Animal-Derived Food Safety, College of Veterinary Medicine, China Agricultural University, Beijing 100193, China; shaoshibeil@cau.edu.cn (S.S); s20213050793@cau.edu.cn (W.S); BS20193050465@cau.edu.cn (Y.B.); suxia@cau.edu.cn (S.Z.); sjz@cau.edu.cn (J.S.); wangzhanhui@cau.edu.cn (Z.W.)

* Correspondence: Correspondence: wenkai@cau.edu.cn

Buffers and solutions

Stock standard solutions (5 g/L) of all analytes were prepared by dissolving an appropriate amount of each standard in N, N-dimethylformamide (DMF). The individual stock solutions were stored at −20 °C in amber glass bottles. Working standards of each analyte were prepared by diluting the stock standard solution in assay buffer and were stored at 4 °C.

The following buffers were used in the enzyme-linked immunosorbent assay (ELISA): (1) coating buffer was 0.05 M carbonate buffer, pH 9.6; (2) blocking buffer consisted of 0.01 M phosphate buffered saline (PBS, pH 7.4) with 0.5% BSA, and 0.01% Tween 20; (3) washing buffer was 0.01 M PBS with 0.05% Tween 20; (4) antibody dilution buffer was 0.01 M PBS containing 0.2% albumin; (5) the enzyme labeled secondary antibody dilution buffer was the antibody dilution buffer containing 5% albumin; (6) substrate was 0.1% 3,3,5,5′-tetramethylbenzidine and 50% H_2O_2 in 0.05 M citrate buffer, pH 4.5; and (7) the stopping reagent was 2 M H_2SO_4.

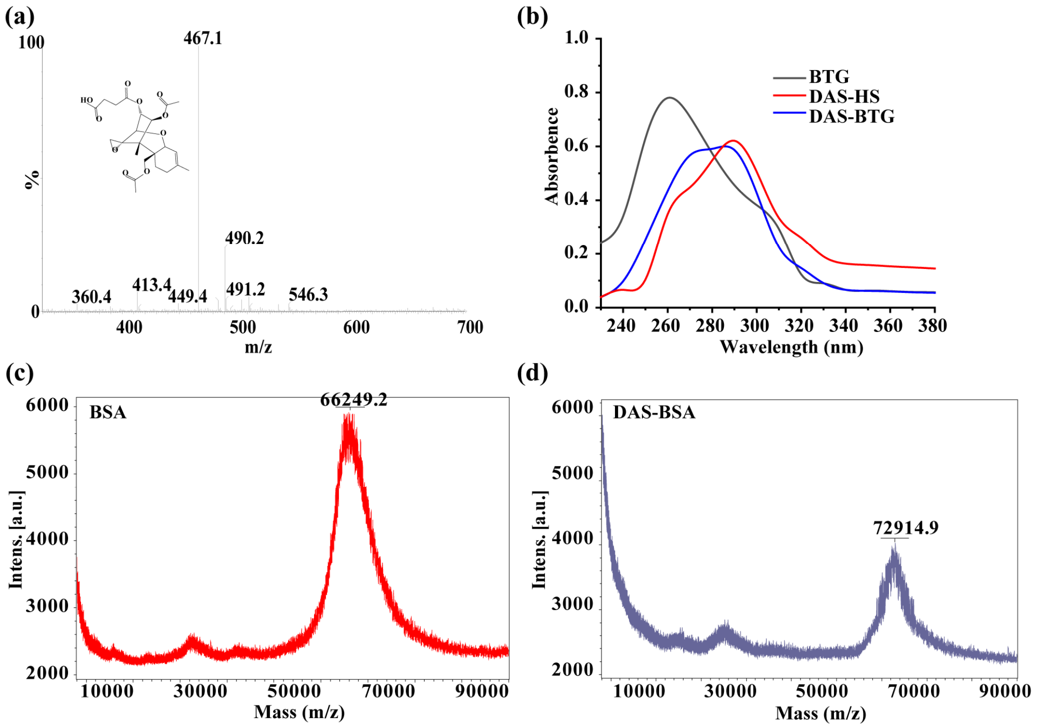

Figure S1. Characterizations of DAS hapten and antigen, (a) mass spectra of hapten DAS-HS, (b) UV-vis spectrum of DAS immunogen, matrix-assisted laser desorption/ionization time of flight mass spectrometry characterizaiton of (c) BSA standard and (d) coating antigen DAS-BSA.

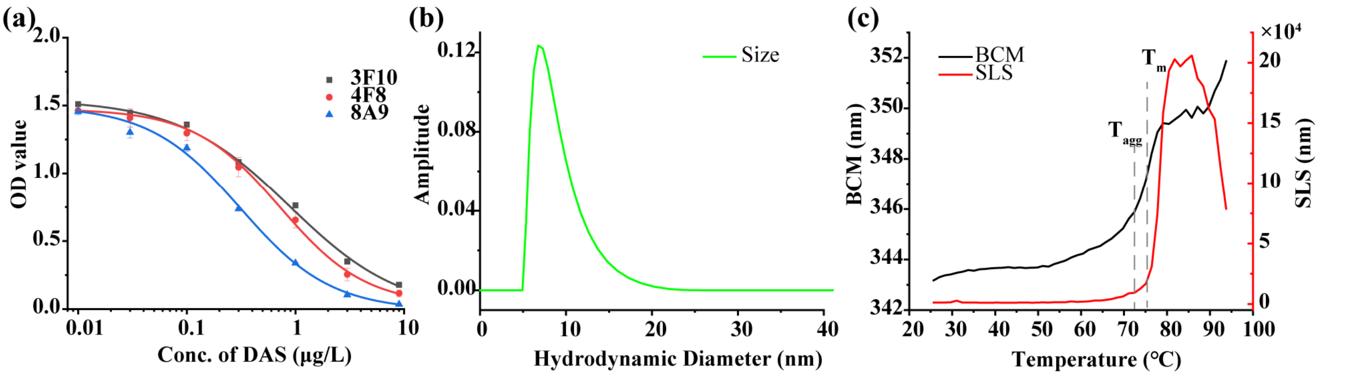

Figure S2. Characterizations of DAS mAb. (a) inhibition curves of different cell lines of DAS, (b) size of mAb in buffer, (b) stability of mAb 8A9.

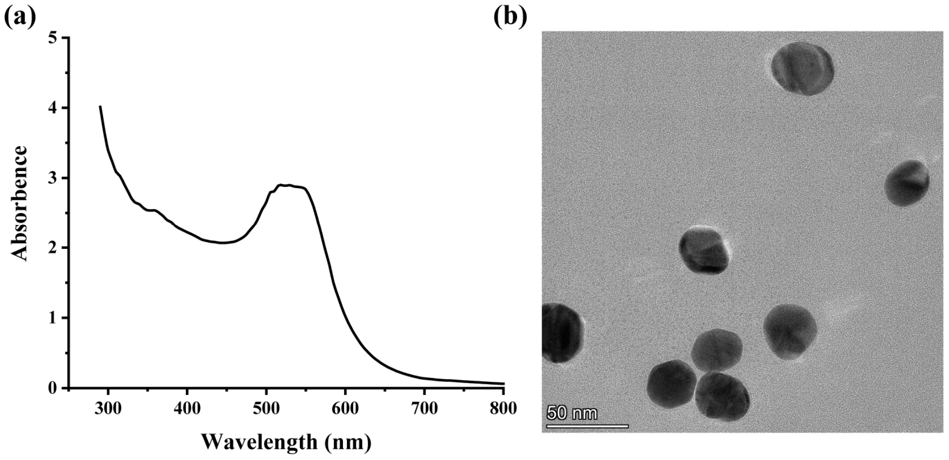

Figure S3. Characterizations of AuNPs, (a) UV-vis, (b) Transmission electron microscopy.

Table S1. Primers used for PCR amplification.

Primer	Sequences（5'-3'）
AS-Linker	PO_4-CTGCAGAAAGCTTGGTGGATCCTA-NH_2
AS04-Primer	TAGGATCCACCAAGCTTTCTGCAG
IgG_1 Primer	GTCGACTCATTTACCAGGAGAGTGGGAGAGG
Lamda Primer	CTC GAG TCC AGA CTC TTC TCC ACA GTG TC
pJB33F	GGCTTTACACTTTATGCTTCCG
pJB33R	CGAGAAAGGAAGGGAAGAAAGC
